# The tryptophan pathway genes of the Sargasso Sea metagenome: new operon structures and the prevalence of non-operon organization

**DOI:** 10.1186/gb-2008-9-1-r20

**Published:** 2008-01-27

**Authors:** Juliana Kagan, Itai Sharon, Oded Beja, Jonathan C Kuhn

**Affiliations:** 1 Faculty of Biology, Technion, Israel Institute of Technology, Haifa, Israel 32000; 2 Computer Science Department, Technion, Israel Institute of Technology, Haifa, Israel 32000

## Abstract

**Background:**

The enormous database of microbial DNA generated from the Sargasso Sea metagenome provides a unique opportunity to locate genes participating in different biosynthetic pathways and to attempt to understand the relationship and evolution of those genes. In this article, an analysis of the Sargasso Sea metagenome is made with respect to the seven genes of the tryptophan pathway.

**Results:**

At least 5% of all the genes that are related to amino acid biosynthesis are tryptophan (*trp*) genes. Many contigs and scaffolds contain whole or split operons that are similar to previously analyzed *trp *gene organizations. Only two scaffolds discovered in this analysis possess a different operon organization of tryptophan pathway genes than those previously known. Many marine organisms lack an operon-type organization of these genes or have mini-operons containing only two *trp *genes. In addition, the *trpB *genes from this search reveal that the dichotomous division between *trpB*_1 and *trpB*_2 also occurs in organisms from the Sargasso Sea. One cluster was found to contain *trpB *sequences that were closely related to each other but distinct from most known *trpB *sequences.

**Conclusion:**

The data show that *trp *genes are widely dispersed within this metagenome. The novel organization of these genes and an unusual group of *trpB*_1 sequences that were found among some of these Sargasso Sea bacteria indicate that there is much to be discovered about both the reason for certain gene orders and the regulation of tryptophan biosynthesis in marine bacteria.

## Abstract 2

An analysis of the seven genes of the tryptophan pathway in the Sargasso Sea metagenome shows that the majority of contigs and scaffolds contain whole or split operons that are similar to previously analyzed trp gene organizations.

## Background

**Figure 1:**
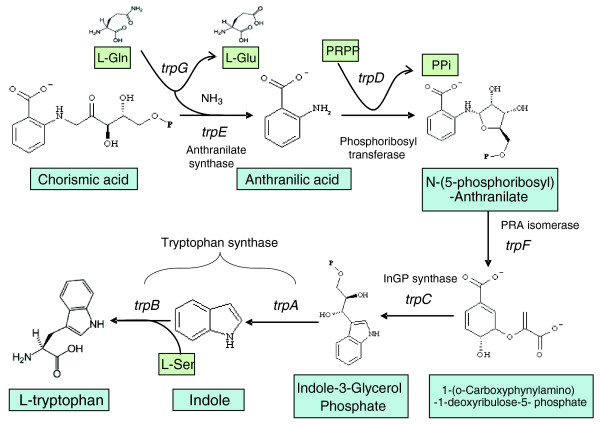
**The biochemical pathway of tryptophan biosynthesis**. The genetic nomenclature for the seven genes that encode the enzymes is that for *Bacillus subtilis*. PR-Anth, N-(5'-phosphoribosyl)-anthranilate; CdRP, 1-(o-carboxy-phenylamino)-1-deoxyribulose-5-phosphate; InGP, indole 3-glycerol phosphate. *trpE *encodes the large aminase subunit of anthranilate synthase; *trpG *encodes for small glutamine binding subunit of anthranilate synthase and catalyzes the glutaminase reaction; *trpD *encodes anthranilate-phosphoribosyl transferase; *trpF *encodes phosphoribosyl-anthranilate isomerase; *trpC *encodes indoleglycerol phosphate synthase; *trpA*, the a subunit of tryptophan synthase which converts InGP to indole; *trpB *encodes the b subunit of tryptophan synthase and converts indole and serine to tryptophan and glyceraldehydes-3-phosphate.

The tryptophan pathway and the organization of the *trp *genes involved in its synthesis have been a model system for many years and these genes continue to receive attention [1,2]. With the availability of extensive DNA sequences, it has been found that *trp *genes are not identically organized in all organisms. The classical structure of the *trp *operon contains genes for all seven catalytic domains in the following order: promoter, *trpE, trpG, trpD*, *trpC*, *trpF*, *trpB *and *trpA*. In some organisms each catalytic domain is encoded by a different gene. As shown in Figure 1, there are seven catalytic domains that carry out the reactions that convert chorismate and L-glutamine to L-tryptophan.

To date, several deviations from the classical structure have been reported. Gene fusion may result in a single polypeptide carrying two or more catalytic domains. The most extreme exception is found in the eukaryote *Euglena *in which a single gene encodes a polypeptide with five catalytic domains [3]. In split operons, the *trp *genes are organized into two or more sub-operons [4]. Other events include gene reshuffling, gene insertions and gene deletions. An analysis of more than 100 genomes showed that the evolution of *trp *operon is both the result of vertical genealogy and lateral gene transfer. It has been found that, if events of lateral gene transfer and paralogy can be sorted out, the vertical transfer of the *trp *genes becomes apparent [4,5].

As a result of the publication of the Sargasso Sea metagenome by Venter *et al*. [6], it may be possible to deduce the evolutionary relationships between the *trp *genes of different marine organisms from the Sargasso Sea. This metagenome is composed of more than one million non-redundant sequences, or reads, that have been estimated to derive from 1,800 different genomes, including 148 phylotypes. These sequences were assembled and scanned for the presence of open reading frames, which were then annotated and analyzed [6]. Overall, more than 1.2 million putative genes were identified, including 37,118 genes for amino acid biosynthesis. Tryptophan pathway genes should be widely represented among these sequences. A vast amount of information about the *trp *genes from various bacterial species exists in the literature and the Sargasso Sea metagenome data should contribute much to our knowledge of the evolution and organizational diversity of these important genes [7], in particular those from a marine environment. Marine bacteria live in an exacting environment that makes selective demands on its inhabitants-in quite a different way to the terrestrial environment.

We have made an extensive search for tryptophan pathway genes within the metagenome data. Our major goal was to determine whether the classical structure of the *trp *operon predominates in marine microorganisms and whether novel structures are present. This information should help us look at questions about the origin of the *trp *genes and the genetic and selective processes that have acted on them including their lateral transfer between different bacterial species

## Results

### Computer search for tryptophan pathway genes

Contigs and scaffolds from the Sargasso Sea metagenome were screened for *trp *genes. The search was run seven times, each using the amino acid sequence of a different *Bacillus subtilis trp *gene. Among contigs and scaffolds, we found 2,926 that had *trp *genes. Of these, 879 contained 2 or more *trp *genes and 2,047 contained only a single *trp *gene. After removing repeats resulting from sequences carrying several *trp *genes, we found 1,928 *trp *genes that were associated with at least one other *trp *gene, which makes it very likely that these are *trp *genes. A total of 4,009 *trp*-like genes were found but some of these might be pseudogenes. That is, a minimum of 5% of all the genes for amino acid biosynthesis (37,118 genes [6]) are *trp*-like genes

The gene order *E-G-D-C-F-B-A *was taken as the prototype for complete operons. For "split-operons", the prototypes used were *E-G-D-C *and *F-B-A*. Table 1 shows the distribution of the contigs for different *trp *genes. The assembly of important scaffolds and contigs (see Table 2) was verified by re-assembling their reads using the SEQUENCHER program version 4.1.2 by Gene Codes Corporation (Ann Arbor, MI, USA). The resulting assembly was found to be consistent with that previously generated by the Celera Assembler [6] The amount of coverage gives an estimate of the frequency of a contig within the population of organisms sampled and was determined for each contig. The results of this search are presented in Table 2. Full and split operons with a classical structure are widely represented.

**Table 1:** Distribution of *trp *gene appearances on scaffolds and contigs in the Sargasso metagenome

Gene	Total number of copies*	With other *trp *genes†	Alone‡
*trpE*	663	277	386
*trpG*	826	396	430
*trpD*	426	278	148
*trpC*	382	153	229
*trpF*	378	235	143
*trpB*	892	408	484
*trpA*	442	215	227
	4,009	879	2,047

* Total number of copies, number of occurrences of the gene in the Sargasso Sea metagenome. † With other *trp *genes, number of occurrences on scaffolds and contigs containing more than one *trp *gene. ‡ Alone, number of occurrences on scaffolds and contigs with no other *trp *genes

**Table 2:** Coverage and gene order of different contigs and scaffolds

Contig/Scaffold	Actual length*	Coverage†	Gene order‡
AACY01037482	5934	10.81	D→C→F→B→A
AACY01011678	5668	10.66	Full operon
CH026811	14769	8.78	Full operon
AACY01096779	10932	8.69	E→G→D→C
AACY01096698	2822	8.51	E→G→D→C
AACY01104100	6690	8.21	E→G→D→C→B→A
AACY01008961	7081	7.36	E→G→D→C
AACY01117014	7301	5.94	E→G→D→C
AACY01092457	4603	4.45	E→G→D→C
AACY01074747	3876	4.26	E→G→PLPDE_IV
AACY01046473	3887	3.96	E→G→D→C
AACY01056517	4373	3.85	E→G→D→C
CH025535	76373	3.72	E→G→D→C→F→B→X→A
AACY01039569	5041	3.45	E→G→D→C
AACY01065695	3747	3.37	E→G→D→C
AACY01088195	7958	3.27	E→G→D→C
CH020599	17648	3.18	G→D→C→F
AACY01010663	3644	3.17	E→G→D→C
CH006047	9399	3.03	Full operon
AACY01056487	4038	2.91	E→G→D→C
CH025058	36,150	2.69	B→A→E→G→D→C
CH025585	10777	2.59	Full operon
CH006071	68188	2.53	Full operon
AACY01110889	4437	2.43	F→(EG)
AACY01063516	4094	2.35	E→G→D→C
AACY01027084	3981	2.21	D→C→F→B→A
AACY01064621	5161	2.02	E→G→D→C
AACY01052709	2451	2.00	E→G→D→C
AACY01079380	1515	1.89	G→C
AACY01015506	2202	1.35	E→G→D→C
CH200199	1879	1.00	E→G→D→C
CH199785	1823	1.00	E→G→D→C
CH174161	1722	1.00	E→G→D→C

*Actual length, number of known nucleotides; †Coverage, average number of reads covering each nucleotide; ‡Gene order, of different contigs and scaffolds.

Table 1 also gives the results for each separate gene. It shows that different genes are not represented with equal frequency: *trpE, trpG *and *trpB *are over-represented. A possible explanation for this is that *trpE *and *trpG *homologues take part in other biochemical pathways such as the pathway for para-amino benzoic acid [8] and have been incorrectly identified as *trp *genes.

A computer search of this type cannot determine the actual enzymatic activity of a particular coding region and this can lead to an over-representation of certain genes. An analysis of the *trpG *and *pabA *genes, which are almost certainly derived from a common source, showed that these cannot be distinguished from one another unless they are associated with an adjacent *trp *gene (for *trpG*) or a *pab *gene (for *pabA*). In the cases where there is no ambiguity as to their identity, it was found that these two genes from the same organism were often more closely related than when they were compared to their counterparts in other organisms (data not shown). An analysis of the *trpE *and *pabB *genes, which also have a common origin, gave similar results. Gene duplication could also cause an apparent over-representation and this is discussed below in reference to the occurrence of the two kinds of *trpB *genes. Genes that encode enzymes that act in more than one pathway and catalyze similar reactions can either appear in searches done on two different pathways or not appear in either search. An example of this phenomenon is the *trpF gene*, which is discussed below.

In order to determine the extent of coverage by this search method, an analysis of the *trpE*, *trpD *and *trpA *genes was made using the genes from the ten different organisms listed in Table 3 as probes. The results of these searches for *trpD *and *trpA *are shown in Table 3.

**Table 3:** Search for *trpD *and *trpA *genes using multiple probes

Species and strain*	matches†	both‡	probe only§	*Bacillus *only¶	% new¥
***trpD***					
*Sulfolobus solfataricus *P2	454	444	10	24	**2**
*Thermoplasma acidophilum *DSM 1728	409	404	5	64	**1**
*Nostoc *sp. PCC 7120	436	430	6	38	**1**
*Thermoanaerobacter tengcongensis *MB4	493	467	26	1	**6**
*Rhodopirellula baltica *SH 1	448	442	6	26	**1**
*Bacteroides fragilis *NCTC 9343	424	419	5	49	**1**
*Corynebacterium jeikeium *K411	443	433	10	35	**2**
*Methanosphaera stadtmanae *DSM 3091	441	433	8	35	**2**
*Neisseria meningitidis *FAM18	474	458	16	10	**3**
*Clostridium kluyveri *DSM 555	492	464	28	4	**6**
**All#**	**514**	**468**	**46**	**0**	**10**
					
***trpA***					
*Sulfolobus solfataricus *P2	222	222	0	241	**0**
*Nostoc *sp. PCC 7120	471	445	26	18	**6**
*Pseudomonas putida *KT2440	498	457	41	6	**9**
*Rhodopirellula baltica *SH1	478	456	22	7	**5**
*Corynebacterium jeikeium *K4111	463	432	31	31	**7**
*Bacteroides fragilis *NCTC 9343	437	431	6	32	**1**
*Clostridium kluyveri *DSM 555	475	443	32	20	**7**
*Thermoplasma acidophilum *DSM 1728	25	25	0	438	**0**
*Neisseria meningitidis *053442	479	452	27	11	**6**
*Leptospira biflexa *serovar Patoc	474	451	23	12	**5**
**All#**	**517**	**463**	**54**	**0**	**12**

* Species and strain, those used to probe the database † Matches, number of genes detected using the specific probe ‡ Both, genes detected by both the specific probe and that from *Bacillus*; § Probe only, those sequences detected by the specific probe but not by that from *Bacillus *¶ *Bacillus *only, those sequences detected by the *Bacillus *probe but not by the specific probe ¥ % new, per cent of new sequences not detected by the *Bacillus *probe # All, the total number of sequences found by all probes; those that were common to *Bacillus *and one or more of the specific probes; the number of genes found with specific probes but not by that from *Bacillus *(new sequences); those found by the *Bacillus *probe but not by the others; the per cent of new sequences, that is the number of new sequences divided by the number of *Bacillus *sequences times 100. The data given in the table are raw data without the elimination of sequences that are somewhat doubtful because in this table we are trying to maximally expand the search parameters.

The analysis of *trpE *sequences is complicated by the concomitant detection of *pabB *sequences. New *trpE *sequences were uncovered and these usually represent about 10% of those detected using the *Bacillus *probe. Using probes of ten species to search for *trpD *led to the discovery of an average of about 3% for each probe. However as many of the new genes will appear in more than one search, only an additional 10% (46/468) of new *trpD *genes were found *in toto*. Table 3 also presents the data for *trpA*, another gene for which little ambiguity is anticipated. That search again led to the discovery of new genes (an average of 4.5% per search) but again the total of new *trpA *genes from the ten probes was only 12% (54/463). Therefore, the coverage provided by the *Bacillus *probes, while not complete, renders a fairly accurate picture of the *trp *genes in the Sargasso Sea metagenome database. We would expect that using more and more probes would be subject to the law of diminishing returns.

### Operon structures

**Figure 2:**
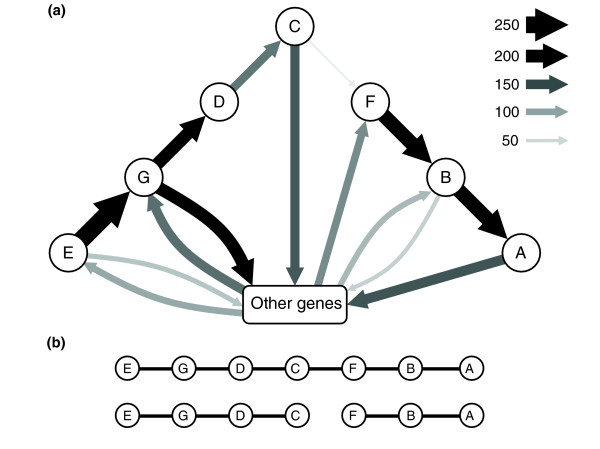
**Distribution of neighboring genes involving at least one *trp *gene**. (a) Each arrow connects neighboring genes, its size and color represents number of pairs found in the Sargasso metagenome (see legend, only pairs observed more than 30 times are shown). Pairs of genes composing the two split operons E→G→D→C and F→B→A are abundant while the pair C→F was rarely found. This may hint that the *trp *genes are usually organized as split operons rather than as full operons. **(b) **The representation of classical full and split trp operons.

Table 4 summarizes the number of scaffolds and contigs that contain several *trp *genes. Some scaffolds have all seven *trp *genes grouped together. The descriptions of several scaffolds of particular interest are presented in Table 5. Eleven of the 24 scaffolds and contigs containing 4 *trp *genes were lacking flanking sequences, and therefore could not be considered as split operons. The other 13 had genes unrelated to the *trp *operon on both ends, or at least after the *trpC *gene (for split operons of the *EGDC *type), and therefore fit the definition of split operons. In the 61 scaffolds and contigs that have three genes together, only 16 contain *trp *genes flanked by those that are unrelated and can be unambiguously denoted as split-operons. The following previously described split-operons were found: *E→G→D→C, F→B→A, F→B→X→A*. Calculations of frequencies of gene pairs (Figure 2) hint that the first two split operons are the most abundant within the Sargasso Sea metagenome, while other organizations, including the classical full operon, are much less abundant. This conclusion may be supported by the very few *C→F *pairs that have been found.

**Table 4:** Number of contigs and scaffolds containing multiple *trp *genes

No. of *trp *genes	No. of contigs and scaffolds
7	8
6	3
5	3
4	24
3	61
2	780
1	2,046

**Table 5:** Description of selected scaffolds

Scaffold	No of *trp *genes in the scaffold	Gene order	Comments
CH027495	6	EGD(CF)B	Lack of *trpA *gene Gap of unsequenced DNA between *trpB *and those genes that are unrelated to *trp *genes may contain gene *trpA*.
CH027608	5	DCFBA	Lack of *trpE *and *trpG *genes. However, the region between *trpD *and genes unrelated to *trp *is missing.
CH011919	5	EGDCBA	Lack of a *trpF *gene There is a gap in the sequence between two neighboring contigs that contain E-G-D-C on the one hand and B-A on the other. Until the connecting pieces are found in both these cases, no decision can be made as to whether the missing genes are separate from the other *trp *genes.
CH005689	5	EGDFB	Lacks both *trpC *and *trpA*. While the absence of *trpC *is not in doubt because *trpD *is adjacent to *trpF*, and on the same contig, *trpA *is probably missing due to the incompleteness of the sequence.
CH026313	4	DCFB	Lack of *trpE trpG *and *trpA *genes. Not definite that this is a split operon because of gaps between *trpD*/*trpB *and their neighboring genes. Moreover the gap between *trpD *and *trpC *challenge the correctness of assembly
AACY01051805 AACY01049273	7	EGDCFBA	*Shewanella oneidensis*, SAR-1 and SAR-2
CH004526 CH004459	Split operon: 4 and 3	EGDC FBXA	One interesting feature of the *trp *genes of *Burkholderia *SAR-1 should be mentioned: in all previously known genomes of *Burkholderia *sp., the split-operons contain *F→B→X→A *where "X" is unrelated to known *trp *genes. The sequence from the Sargasso Sea metagenome of SAR-1 *Burkholderia*-like sequences contains an *F→X→A *split operon. The computer program used by Venter and colleagues failed to identify a *trpB *gene within the sequence. However when a search was made using the *Burkholderia trpB *sequence as a probe, a *trpB *gene was detected between *trpF *and X, as is true for all other *Burkholderia *species and there were no non-*trp *genes between *trpF *and *trpB*.

**Figure 3:**
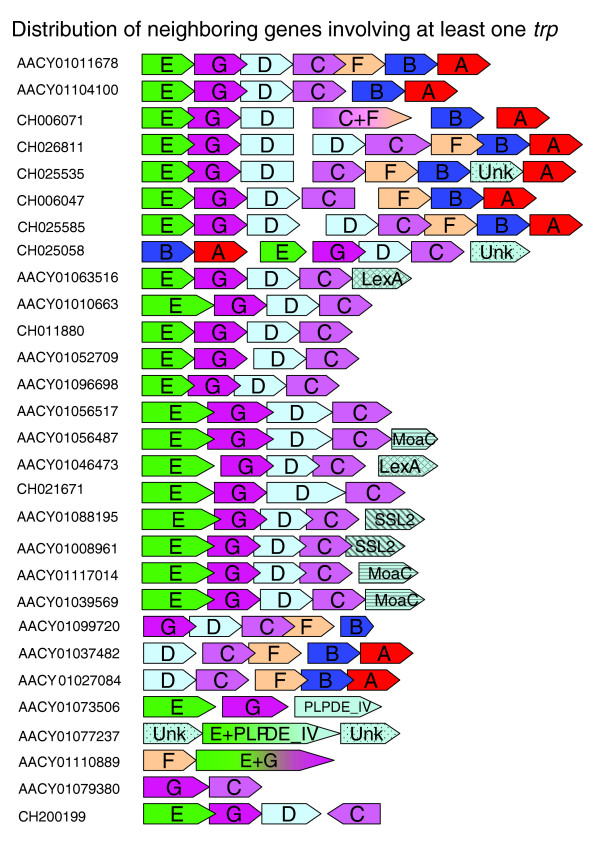
**Alignment of *trp *sequences from different contigs and scaffolds**. The following abbreviations are used: E, *trpE*; G, *trpG *(or sequences with a high similarity to *pabA*); C, *trpC*; D, *trpD*; F, *trpF*; B, *trpB*; A, *trpA*; Unk, an ORF with unknown function; *truA*, the tRNA pseudouridine synthase; *moaC*, a protein related to the molybdenum cofactor; *SSL22*, DNA or RNA helicases of superfamily II; *lexA*, the SOS-response transcriptional repressor.

As illustrated in Figure 3, most of the complete and incomplete *trp *gene clusters maintain the structure of the prototype *trp *operon. All genes within these clusters have the same direction of transcription and the same gene order. Two of the split operons, [GenBank: AACY01080023] and [GenBank: AACY01120345], seem to be from the genome of *Burkholderia *SAR-1, while two full operons described in Table 5 seem to come from *Shewanella *SAR 1 and 2. As the sequences of these do not differ from those found earlier for those organism and the probable source of these is a filter contamination as has been stated in several papers [9,10] they were not taken into account in our calculations.

Two contigs show a different type of organization than that generally found in bacteria. In one contig [GenBank: AACY01110889] *trpF *is followed by a gene that is a fusion between *trpE *and *trpG*. This contig is a part of a scaffold, [GenBank: CH022404], which shows no similarity to any known bacterium with regard to *trpE *and *trpG*. While the fusion of *trpG *and *trpE *has been found in bacteria such as *Legionella pneumophila, Rhodopseudomonas palustris, Thermomonospora fusca, Anabaena sp*. and *Nostoc punctiforme*, none of them contain the gene order *F-(E-G)*. However, the gene order *trpF*-*trpE-trpG *has been found in some *Archaea *such as *Halobacterium sp., Methanosarcina barkeri *and *Ferroplasma acidarmanus*, but in these species *trpE *and *trpG *are separate genes. In a second contig [GenBank: AACY01079380] the gene order *trpG-trpC *has been observed. This gene order has already been described for *Archaea *such as *Thermoplasma acidophilum, Thermoplasma volcanium, Ferroplasma acidarmanus and Sulfolobus solfataricus *[4].

The order of adjacent *trp *genes within two scaffolds, [GenBank: CH025058] (gene order: *B-A-E-G-D-C*) and [GenBank: AACY01110889] (gene order: *F-(EG*)) are entirely novel and have not been observed to date. Both have a relatively high coverage in the database, which confirms the importance and abundance of these gene orders in marine populations. An analysis of other, non-*trp *genes within these scaffolds failed to reveal any significant similarity between them and known genomes.

**Figure 4:**
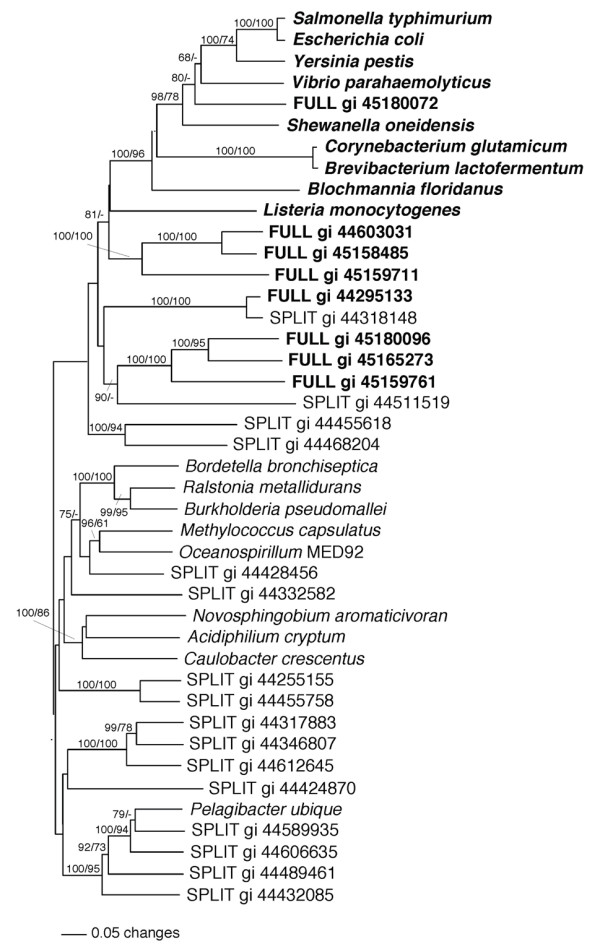
**Phylogenetic analysis of scaffolds and contigs containing whole and complete operons**. The concatenated amino acid sequences from genes *trpE*, *trpG*, *trpD*, and *trpC *were used to analyze the relationships among both known species and those from the Sargasso Sea metagenome. Full operons are written in bold whereas split operons are not.

A phylogenetic analysis of some of these complete and split operons was made against operons from known organisms. The results are presented in Figure 4. All the full operons are much more related to the full operons of known organisms than they are to the split operons of other known species. The figure also shows that most of the split operons are grouped with split operons from known organisms. The four exceptions to this rule are probably due to incomplete sequences and these are likely to be full operons. This analysis also supports our hypothesis that split operons are more prevalent than full operons (Figure 2) in the Sargasso Sea metagenome

### Non-operon organization

As shown in Table 4, 70% of the contigs and scaffolds detected have a single *trp *gene. Those with two *trp *genes are also very prevalent (26%) even though some of these are probably partial segments of larger operons. As shown in Table 6, 133 scaffolds and contigs carry one or two *trp *genes enclosed between non-*trp *genes. While *trpE *and *trpG *may be overrepresented due to the existence of homologous genes as mentioned above, other *trp *genes are also observed in a "detached" manner. This indicates that the *trp *genes of marine organisms are frequently detached or occur as pairs.

**Table 6:** Frequency of scaffolds and contigs containing unusual organizations of trp genes.

Gene order (enclosed)*	No of occurences†	Gene order (partial) ‡	No of occurences
X→E→X	16	E→X	55
X→G→X	42	X→G	88
X→D→X	3	G→X	108
X→C→X	5	X→D	16
X→F→X	2	D→X	8
X→B→X	7	X→C	16
X→A→X	5	F→X	6
X→B→A(→X)	9	X→B	69
(X→) E→G→X	44	B→X	49
		X→A	16
**Total**	**133**	**Total**	**431**

* Gene order (enclosed), organizations of one and two trp operons enclosed between non-trp genes; † Number of occurrences, number of contigs and scaffolds carrying the organization; ‡ Gene order (partial), pairs of *trp *and non-*trp *genes that are inconsistent with classical organization.

The existence of pairs of *trp *genes makes good sense biochemically. Anthranilate synthase is composed of an equal number of *trpE *and *trpD *encoded subunits. Tryptophan synthase contains two subunits each of the polypeptides from the *trpA *and *trpB *genes. The *trpG *when unfused to *trpE *or *trpD *leads to a polypeptide also found in equimolar amounts to those from *trpE *and *trpD*. Organizing these specific genes in pairs would seem to ensure that they are transcribed together and render the proper amounts of the translation products.

The occurrence of detached *trp *genes is apparently an adaptation to the particular environment in which marine organisms are found. Most of the bacteria previously analyzed probably encounter periods of feast and famine with regard to tryptophan. Therefore they need to respond to external conditions that vary. The existence of transport systems for concentrating externally found tryptophan and the organization of the *trp *biosynthetic genes into operons almost certainly reflect their environmental challenges. In contrast, marine organisms exist in a rather constant environment with respect to tryptophan. It is unlikely that tryptophan from external sources is available and this amino acid must be synthesized entirely within the bacterial cell. The main regulation of the pathway is expected to be at the level of feedback inhibition and it is probable that *trp *gene expression is constitutive rather than controlled by the mechanism of repression-derepression. The level of expression of a detached *trp *gene can be controlled simply by modifying the strength of the associated promoter. A *trp *repressor or repressors and attenuation become superfluous under such circumstances. This should extend to most or all of the other genes involved in amino acid biosynthesis. Therefore axenic cultures of some of these marine organisms are eagerly awaited.

### Conserved non-*trp *flanking genes

Another way of examining the evolution of the *trp *genes and the relationships between various species is the analysis of genes not involved in tryptophan biosynthesis that either neighbor the *trp *genes or are inserted between them. Xie and colleagues have reported that *trpF*, *trpB *and *trpA *in split-pathway operons are flanked by conserved genes that are unrelated to tryptophan biosynthesis [4]. They have found genes that encode the β-subunit of acetyl-coenzymeA-carboxylase (*accD*), folylpolyglutamate synthase/dihydrofolate synthase (*folC*), fimbria V protein (*lysM*) and the tRNA pseudouridine synthase (*truA*). In most cases the genes *accD *and *folC *follow *trpA*. For the *Thiobacillus-Pseudomonas*-*Azotobacter *cluster and others, the *trpF*-*trpB*-*trpA *operon is flanked on the *trpF *side by *lysM *and *truA*. The presence of particular genes appearing near those of *trp *was examined using the Sargasso Sea metagenome data and the results of this analysis are shown in Table 7.

**Table 7:** Genes flanking the *trp *operon

Gene	Number of times in the metagenome	Percent found near *trp *genes
*TruA*	30	93% are adjacent and before *trpF*
*AccD*	53	86.8% are adjacent and after *trpA* 9.4% are adjacent and after *trpB*; *trpA *is elsewhere 3.8% are adjacent and after *trpF*; *trpB *and *A *are absent
*FolC*	13	77% occur as *trpA-accD-folC* 23% occur in the order of *trpB-accD-folC*
*PyrF*	60	77% are before *trpF *in split operons 23% are before *trpB*
*LexA*	92	100% are adjacent and after *trpC *when *trpF *is elsewhere
*MoaC*	25	100% neighbor and are after *trpC*
*PLPDE_IV*	21	57% adjacent and after *trpE* 38% adjacent and after *trpG*

The first three rows of Table 7 confirm previous publications. In addition, four other genes, not previously noted, were found with high frequencies near the *trp *genes of the Sargasso Sea metagenome: *pyrF *(orotidine-5'-phosphate decarboxylase), *lexA *(the SOS-response transcriptional repressor), *moaC *(a protein related to the molybdenum cofactor) and *PLPDE_IV *(the class of amino acid aminotransferases). It should be mentioned that *PLPDE_IV *is the only gene, besides *aroG *and *aroH *(see below), found near the *trp *genes that can be logically connected to tryptophan biosynthesis. This class of amino-transferases includes some D-amino acid transferases, pyridoxal-5-phosphate-dependent enzymes such as tryptophanase, and others. If in fact the cell is able to use D-tryptophan as a source of L-tryptophan via a D-amino acid transferase, then the inclusion of a gene encoding such an activity among the *trp *genes would make sense as this gene would undergo derepression in coordination with those involved in L-tryptophan biosynthesis.

It is clear that specific neighboring genes are very prevalent when a split *trp *operon occurs. It seems unlikely that the same event has occurred many times: strains with these particular flanking genes are most likely derived from a common ancestor.

### Analysis of *trpB *genes

Surprisingly, it has been found that a significant number of organisms possess more than one *trpB *gene encoding the β-chain of tryptophan synthase. Usually, but not always, the 'extra' gene is unlinked to the *trpA *gene encoding the α chain of this enzyme. These extra *trpB *genes belong to a distinct subgroup encoding the β-chain which is termed *trpB*_2. This had been recognized in the COGs database as "alternative tryptophan synthase" - COG_1350 _[11] while the major group is denoted as *trpB*_1 and includes the well-studied polypeptides from such organisms as *Escherichia coli, Salmonella typhimurium *and *Bacillus subtilis*. The minor *trpB*_2 group includes mostly, but not exclusively, archaeal species. The evolution and properties of *trpB*_2, have been analyzed and discussed in a number of recent articles [12-15].

The 3-dimensional structure of tryptophan synthase from *Salmonella typhimurium *has been elucidated by X-ray crystallography to a resolution of 2.5 angstroms [16]. The enzyme is a αββα complex which forms an internal hydrophobic tunnel into which indole, produced by the a subunit, enters and then reaches the active site of the b subunit. The α monomers and β dimers contact one another via a highly specific mechanism of recognition. In addition, the genes encoding these two subunits are almost always closely linked and their expression is frequently translationally coupled [17,18].

**Figure 5:**
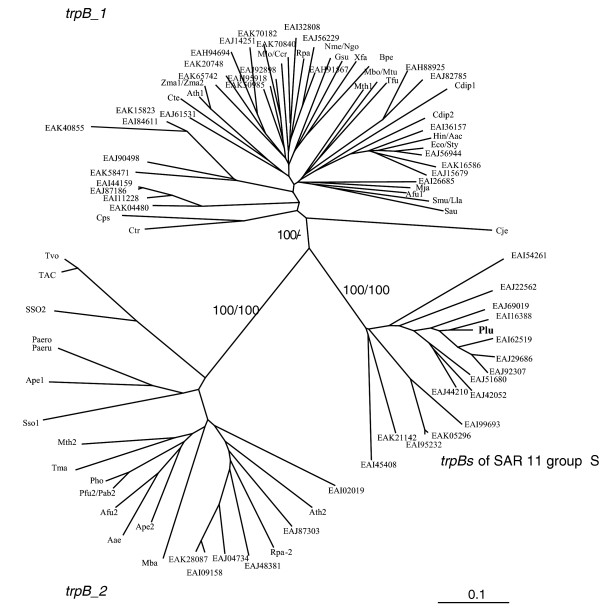
**Representation of Sargasso metagenome *trpB *sequences and those from known bacteria with respect to genetic distance**. 40 representatives from *trpB *sequences analyzed here were chosen for this analysis. As can be seen, the constructed tree shows two distinct groups; however a third group appears which consists of only environmental sequences and the Ple (*Pelagibacter ubique *(Candidatus)) sequence. The abbreviation of *trpB *genes from known bacteria are listed in Table 8. For the environmental *trpB *sequences abbreviation the NCBI accession numbers were taken. Bootstraps for the main groups are shown.

The data collected from the Sargasso Sea metagenome were examined to determine whether the *trpB *sequences from the Sargasso Sea differ from those of known organisms and whether both *trpB*_1 and *trpB*_2 exist in this sample. When a phylogenetic analysis of *trpB *genes found in the present survey was conducted, it was found that the majority of these (Figure 5) fall into the *trpB*_1 group while a few *trpB*_2 genes also occur. Among the *trpB*_1 genes, one cluster is quite distinct and probably split off from major type at a relatively early stage. Genes in this cluster have a high similarity to the marine bacterium *Pelagibacter ubique *(Candidatus) HTCC1062 (SAR11) and the sequence identity of these to *P. ubique *at the amino acid level was between 64% and 87% while the genes neighboring some of these *trpB*s showed an even higher identity to their counterparts from SAR11. One of the most remarkable features of *P. ubique *is its extremely small genome that lacks any pseudogenes or recent gene duplications. It has only one copy of *trpB*, and therefore it can be concluded that this gene must be functional in tryptophan biosynthesis and not a pseudogene. *P. ubique *contains two split operons: *trpE-trpG-trpD-trpC *and *trpF*-*trpB*-*trpA*. The gene order of the neighboring, non-related *trp *genes of the second split operon is: (gene not mentioned above) *himD-pyrF-trpF-trpB-trpA-accD-folC*. The *himD *gene encodes a sequence-specific DNA-binding transcriptional activator. Comparison of the gene order between contigs containing SAR11-like *trpB *from the Sargasso Sea metagenome showed that most of the contigs have a gene order that is similar to SAR11. Three of 37 contigs lack *trpF *and 2 contigs contain only a *trpB *gene flanked by genes unrelated to *trp *and which are similar in sequence and order to that of SAR11. This indicates that most or all of these *trpB *genes are part of the SAR11 group. Since the *trpB *of SAR11 is more closely related to *trpB_1 *than to *trpB_2 *[19], it seems that the genes from this particular cluster should probably be considered to be of the *trpB*_1 type.

## Discussion

The tryptophan operon of bacteria has been studied for more than 50 years and its structure and regulation are known for many terrestrial organisms that can be grown in laboratory culture. With the explosive expansion of genomics during the last decade and the data thus generated, many *trp *sequences from both known and unknown marine species have become available. This provides an excellent opportunity for expanding our knowledge about the ways in which different organisms, particularly marine bacteria, have organized these genes. In the present research, *trp *pathway genes within the Sargasso Sea database were retrieved by BLAST analysis using known *trp *protein sequences. It was found that *trp *genes account for about 5% of all genes that were previously identified as genes for amino acid synthesis in the Sargasso Sea metagenome. In almost all cases in which the *trp *genes form an operon, the order and direction of transcription of the *trp *genes are similar to familiar prototypes. The reason for this conservation remains unknown. This might be explained in part by an advantage conferred when genes whose products form complexes are adjacent to one another and translational coupling occurs. Of the 85 contigs and scaffolds that contain three or four *trp *genes, only 29 could be unambiguously defined as containing split pathway operons. The following already known orders of split operons were found: *E→G→D→C, F→B→A*. In addition, we have found evidence for completely dispersed *trp *genes in the form of isolated and pairs of genes.

Since these marine organisms survive and grow in a very different environment from those organisms previously studied, they are likely to have been genetically separated from them and to have evolved to solve the particular regulatory problems that exist in their environment. It was expected that some marine bacteria would exhibit novel organizations of these genes and such organizations were in fact found. Among the *trp *genes organized into operon structures, most resemble examples already discovered. In addition, two previously unknown groupings were uncovered in the present search. However a notable quantity of genes that were either detached or in mini-operons containing only two *trp *genes was discovered. Novel organizations of the *trp *genes probably arise from adaptations to the marine environment and it is likely that some marine bacteria will have unusual regulatory features. Such features can only be elucidated when these organisms become amenable to axenic culture. Cloning and expressing these genes in the laboratory from those organisms that cannot yet be cultured may however provide a partial regulatory picture. In this regard, a search for genes related to the *trpR *gene of *Escherichia coli *(the gene that encodes the tryptophan repressor) in the Sargasso Sea metagenome was performed. This search failed to reveal any significant *trpR *homologs. This is not surprising, because regulatory circuits undoubtedly arise later than the genes for biosynthesis and are adaptations to specific environments.

Genes with unknown function have been previously found to be inserted within the *trp *operon [4]. Such genes were found between *trpB *and *trpA *in one contig from the Sargasso Sea metagenome, a location already observed for some species of *Flavobacterium *and *Burkholderia*. Another contig carried such a gene between *trpF *and *trpB*. While the reason for the presence of these non-*trp *genes is unclear and the possibility exists that they are simply morons [20], it is possible that they actually participate in tryptophan biosynthesis. That is, these genes may not be essential for tryptophan synthesis but rather aid it by increasing the catalysis of one of the enzymes or by being involved in complex formation. Even a very small advantage is expected to be of great importance for the survival of an organism in an oligotrophic environment such as that of the Sargasso Sea.

One should keep in mind that the arrangement of genes in operon confers both advantages and disadvantages. The most obvious advantage is that genes with similar function are transcribed together. The greatest disadvantage is that, unless some further level of regulation exists (differences in the amounts of mRNA or its stability, the strength of ribosomal binding sites, and so on), the amount of the polypeptides from these genes will be the same even though the resultant enzymes may have different catalytic rates [21]. The ones with slower rates will be the limiting factor. As a result, when the genes are transcribed together, an excess of some enzymes is likely to occur. However, the amount of mRNA and polypeptide synthesis is only one aspect of the control of the tryptophan pathway. Besides these, there are two other levels of control that affect the amount of tryptophan synthesis within the cell. The first of these is feed-back inhibition which influences the activity of the first two reactions [22], and thereby the amount of metabolites flowing through the pathway. The second is the formation of multi-enzyme complexes that greatly increases the catalytic efficiency of the various reactions. In complexes, the product of one reaction can be used directly by the next enzyme and the concentration of the substrate in the vicinity of the second enzyme is much higher than would occur were the two enzymes separate. Examples of such complexes are *trpE*-*trpD *(*trpG*) and *trpA*-*trpB *and the *trpC*-*trpF *gene fusion in *Escherichia coli*. In addition, one polypeptide can greatly enhance the activity of a second when a complex is formed (for example, in the *trpA*-*trpB *heterotetramer, αββα from *Escherichia coli *[23-25].

Different solutions to the problems of optimal synthesis of tryptophan and the regulation of *trp *gene expression would not be surprising since this amino acid is one of the most expensive in chemical terms. One solution might be to organize the *trp *genes in a different manner; another would be the creation of *trp *gene fusions. Both of these have been observed. Our analysis uncovered some known gene fusions, *E-G *[GenBank: AACY01100727] and *C-F *[GenBank: AACY01022048] and two novel fusion of a *trp *gene with a gene unrelated to the *trp *genes: *E-PLPDE_IV *[GenBank: AACY01077237] and *F-TruA *[GenBank: AACY01600616]. All of above indicate that there is quite a lot of genetic diversity among marine bacteria.

It was found that several specific genes are often neighbors of the *trp *genes of marine microorganisms. When present in contigs, *lexA*, *pyrF *and *moaC *were always placed after *trpC*. This may be a general phenomenon but our information is still too scanty to allow a definite conclusion to be drawn. Similarity in gene order is usually taken to indicate an evolutionary relationship between such segments. Of particular interest was the observation that in 3 cases *aroH *or *aroG *occur adjacent to *trpA*. For these examples, the distance between the end of *trpA *and the ensuing *aro *gene is 3, 18, or 20 base pairs, which makes it very likely that the two genes are expressed together. The synthesis and activity of the enzyme they encode, DAHP synthase, is involved in the synthesis of a precursor of chorismic acid and this *aro *gene is often regulated by the level of tryptophan. Therefore such an arrangement might make sense.

Since there is more than one kind of *trpB *gene, a comparison was made of amino acid sequences of *trpB *genes from the Sargasso Sea metagenome with those from known organisms. The majority of the metagenomic *trpB *sequences detected fall into the *trpB*_1 group while some others were related to the *trpB*_2 group. One cluster containing a number of *trpB*_1 sequences is quite distant from the usual type and has a high similarity to that of *Pelagibacter ubique *(Candidatus) HTCC1062 (SAR11). This cluster probably diverged rather early from the major *trpB*_1 line.

## Conclusion

The present analysis has revealed that tryptophan genes are rather frequent within the Sargasso Sea metagenome. All *trp *genes that were found have enough similarity to COGs to be recognized. This seems to indicate, but does not prove, that all have come from a common ancestor. However, additional genes for tryptophan biosynthesis may exist which we were unable to detect with the probes employed. In this regard, it has been reported [26] that some organisms indeed lack a recognizable *trpF *in their genomes but are capable of growing without external tryptophan. A gene whose sequence is not homologous to known *trpF*s but whose product catalyzes this reaction has in fact been found in *Streptomyces coelicolor *A3 and *Mycobacterium tuberculosis *HR37Rv [26]. This *trpF *gene is an example of reticulate evolution because it can catalyze reactions in both the histidine and tryptophan pathways [27,28]. A BLAST search with the amino acid sequence of the *trp*F gene from *Streptomyces coelicolor *A3 gene (SCO2050) against the Sargasso Sea metagenome data showed more than 500 hits that can be identified as *hisA *proteins. Thus, only a functional analysis of these environmental sequences can prove whether they can take part in both pathways or not. The fact that a group of marine *trpB*_1 sequences are similar to one another but quite distant from the major *trpB*_1 group supports the idea that there may be *trp *genes that are not recognized as such by those sequences presently known.

While *trp *operons, both complete and split, exist in marine bacteria, many *trp *genes are no longer found in that framework. In contrast to most terrestrial bacteria, the operon structure is not used for the *trp *genes in some of marine origin. There are mini-operons of 2 genes in many cases (Table 5) and also an even more frequent occurrence of single *trp *genes. It is of course an open question whether what we observe is the result of the breakup of an original operon structure or that the *trp *operons at present have arisen from these unlinked genes. Since the marine environment is very exacting and selective, it is certain that organisms lacking an operon structure for the *trp *genes have found an evolutionary advantage in the organization of the *trp *genes that they possess. It should be mentioned that in *Escherichia coli *and *Salmonella*, about 50% of the genes encoding polypeptides involved in amino acid synthesis are separate although their *trp *genes are not. On the basis of our results in which novel *trp *gene orders were found, it appears likely that further studies of the *trp *genes and their regulation and organization will provide many future surprises.

## Materials and methods

### Analysis of Sargasso Sea metagenome database

Amino acid sequences with homology to each *trp *catalytic domain were obtained from an NCBI BLAST search of the Sargasso Sea metagenome database [29]. The amino acid sequences from *Bacillus subtilis *of each pathway catalytic domain were used as query entries for protein BLAST. *Bacillus *proteins were chosen as a starting point for the search because the catalytic domains are encoded by separate genes. In *Bacillus *six genes, except *trpG*, are organized into one operon and have been intensively studied at the level of DNA, RNA and protein levels [30-32]. For the *trpB*_2 search, the sequence of *Chlorobium tepidum *CT0192 (Q8KF11) was used. The list of *trp *genes has been generated in several steps. First, BLAST searches of *trp *genes against the Sargasso Sea metagenome has been performed, using an e-value threshold of 1e-5. For cross validation, both peptide and DNA sequence databases were searched and the results were compared. While 95% of the ORFs were identified in both searches, some were discovered only once. In such cases a manual check of the results has been performed. In addition, genes that are homologous to *trp *genes (*PabA*, *PabB*, *PhzA *and *PhzB*) were used to remove misclassified *trpE *and *trpG *genes. As a result, a list of contigs containing *trp *genes was created. Redundant contigs were removed based on BLAST searches with a 95% identity threshold. In the last step, contigs that belong to the same scaffolds were identified and treated. The results of the above semi-automatic process were validated by large-scale manual examinations.

In order to assemble a contig, Venter and colleagues used the Celera Assembler [6]. To validate the Sargasso Sea scaffolds the following procedure was performed. First, all singleton reads composing each scaffold were retrieved by conducting a BLASTn search for the scaffolds against the Sargasso Sea reads. Next, the SEQUENCHER program (Gene Codes Corporation) was used for re-assembling the reads and the results were compared to each original scaffold for validation. No significant differences between the assemblies of the Celera Assembler and SEQUENCHER were found.

Coverage was calculated by recruiting reads from Sargasso Sea using BLAST, considering only reads with 90% and higher identity to the scaffold and at least 80% of the read taking part in the alignment. These parameters are rather stringent, but give a good indication with respect to the distribution of each scaffold.

### Phylogenetic analysis

Amino acid sequences of many *trpB *genes were used to analyze the phylogenetic relationships between different environmental samples. Only genes encoding more than 251 amino acids were analyzed. The alignment was done using the ClustalW program [33]. Neighbor joining (NJ) and maximum parsimony (MP) analyses were conducted on protein data sets using version 4.0b10 of PAUP [34]. Default parameters were used in all analyses. Bootstrap resampling of NJ (1000 replicates) and MP (1000 replicates) trees were performed in all analyses to evaluate the reliability of the inferred topologies. The resultant trees were viewed through the TreeView (Win32) program [35]. To understand the relationship between the sub-families each was analyzed both by comparing one group against the others and to representative *trpB *gene sequences that exist in the NCBI database.

## Abbreviations

MP, Maximum Parsimony analysis; NJ, Neighbor Joining analysis; ORF, Open Reading Frame; SSM, Sargasso Sea Metagenome; trp, Tryptophan. Additionally Table 8 lists the species names used and their abbreviations

**Table 8:** List of species names and their abbreviations

Species name	Abbreviation used	NCBI number
*Aeropyrum pernix*	Ape-1	Q9Y8T5
*Aeropyrum pernix*	Ape-2	Q9Y9H2
*Aquifex aeolicus*	Aae	O67409
*Arabidopsis thaliana*	Ath-1	P14671
*Arabidopsis thaliana*	Ath-2	BAB10143
*Archaeoglobus fulgidus*	Afu-1	O28672
*Archaeoglobus fulgidus*	Afu-2	O29028
*Bordetella pertussis*	Bpe	NP_882102
*Campylobacter jejuni*	Cje	CAL34499
*Pelagibacter ubique *(Candidatus) HTCC1062	Ple	YP_265913
*Chlamydia psittaci*	Cps	Q822W9
*Chlorobium tepidum*	Cte	Q8KF11
*Corynebacterium diphtheriae*	Cdip-1	NP_940652
*Corynebacterium diphtheriae*	Cdip-2	NP_940660
*Escherichia coli*	Eco	P0A879
*Geobacter sulfurreducens*	Gsu	AAT73768
*Haemophilus influenzae*	Hin	P43760
*Lactococcus lactis*	Lla	Q01998
*Legionella pneumophila*	Lpn	CAH15507
*Mesorhizobium loti*	Mlo	NP_105798
*Methanobacterium thermoautotrophicum*	Mth-1	O27696
*Methanobacterium thermoautotrophicum*	Mth-2	O27520
*Methanococcus jannaschii*	Mja	Q60179
*Methanosarcina barkeri*	Mba	AAZ72487
*Mycobacterium bovis*	Mbo	NP_855291
*Mycobacterium tuberculosis*	Mtu	P66984
*Neisseria gonorrhoeae*	Ngo	Q84GJ9
*Neisseria meningitides*	Nme	AAF41116
*Pyrobaculum aerophilum*	Paero	Q8ZV44
*Pyrococcus abyssi*	Pab-2	Q9V150
*Pyrococcus furiosus*	Pfu-2	Q8U0J5
*Pyrococcus horikoshii*	Pho	NP_143439
*Rhodopseudomonas palustris*	Rpa-1	YP_779393
*Salmonella typhimurium*	Sty	NP_460685
*Staphylococcus aureus*	Sau	BAB42464
*Streptococcus mutans*	Smu	NP_720974
*Sulfolobus solfataricus*	Sso-1	P50383
*Sulfolobus solfataricus*	Sso-2	AAK41396
*Thermomonospora fusca*	Tfu	YP_289226
*Thermoplasma acidophilum*	Tac	Q9HKD2
*Thermoplasma volcanium*	Tvo	NP_111450
*Thermotoga maritime*	Tma	Q9WZ09
*Xylella fastidiosa*	Xfa	C82688
*Zea mays*	Zma-1	P43284
*Zea mays*	Zma-2	P43283

## Authors' contributions

JKa and JKu conceived the idea for this analysis and JKu contributed the main guidelines and concepts of the article. JKa performed manual check of the results and was responsible for data organization. IS performed bioinformatics involved in this study. OB performed the phylogenetic analysis. JKu and JKa prepared the initial manuscript. All authors participated in the analysis of the data. All authors have read and approved the final article.
